# Analysis of microbial differences in amniotic fluid between advanced and normal age pregnant women

**DOI:** 10.1186/s12967-021-02996-y

**Published:** 2021-07-27

**Authors:** Ya Wang, Chunyu Luo, Yiwei Cheng, Li Li, Dong Liang, Ping Hu, Zhengfeng Xu

**Affiliations:** 1 Department of Prenatal Diagnosis, Obstetrics and Gynecology Hospital Affiliated to Nanjing Medical University, Nanjing Maternity and Child Health Care Hospital, Nanjing, 210004 China; 2 Nanjing Jiangbei New Aera Biopharmaceutical Public Service, Platform No.3-1 Xinjinhu Road, Zhongdan Life Science and Ecology Industrial Park, Nanjing, 210000 China

**Keywords:** Amniotic fluid, Microorganisms, 16S rRNA gene sequencing, Proteomics

## To the Editor

Amniotic fluid (AF) has been considered sterile for nearly a century because no microorganisms were identified by traditional culture methods [1]. However, this opinion paradigm has been challenged by recent studies based on culture-independent sequencing techniques [2–4] and AF proteomics [5]. Recently, a conclusion that no microorganisms were present in the mid-trimester AF of healthy pregnancies was reached using culture-independent sequencing techniques [6] and seemed to settle the argument [7]. However, it could not explain why non-human proteins were identified in normal human AF supernatants [8] and why microbial exposure primes fetal immune cells in fetal tissues during fetal development [9].

Intra-amniotic infection caused by microbial invasion of the amniotic cavity (MIAC) was associated with adverse pregnancy outcomes, when the bacteria were at high concentrations [10]. This may be a reason why people think that AF is sterile and that bacteria in AF are abnormal because of their negative effects.

Meanwhile, we could identify bacterial proteins in the AF proteomics database that were consistent with the results of 16S ribosomal RNA (rRNA) gene sequencing. Nine amniotic fluid samples were collected from 9 pregnant women and the pregnancy outcomes of the participants were followed. No bacteria were found by cultivation, but a sparse microbial presence was found by proteomics analysis and 16S rRNA gene sequencing approach. The 148 microbes found in the human AF proteomics database were consistent with the microbes found in the 16S rRNA gene sequencing database. The species composition and the structure of communities in the normal age (<  35 years old) and advanced maternal age (AMA) (>  35 years old) pregnancies differed significantly. However, all of the newborns were healthy and had no allergic reactions.

Proteomics analysis has identified nearly 2000 proteins in AF during the past 20 years [11], including many non-human proteins [8]. In a recent study, 7 normal AF samples were used to generate human AF proteomes, which were divided into 4 groups: original proteins, bound proteins, flow-through proteins, and iTRAQ-labeled individual/mixed digested peptides [11]. We reanalyzed the human AF protein database and found that a part of the non-human proteins were derived from microorganisms. A total of 148 microbial-associated proteins in the normal human AF proteome, which could potentially play important roles in cellular/metabolic processes and binding/catalytic activity (Additional file 1). Notably, all of the microbial-associated proteins were present at a low level in human AF. These data suggest that there may be low concentrations of microorganisms in normal human AF samples.

To test our hypothesis, we conducted a study to investigate the presence of microorganisms in the mid-trimester AF of 9 women [4 normal age [normal] and 5 advanced maternal age [AMA]; advanced maternal age is generally defined as age above 35 years at the time of delivery [12, 13]) by cultivation and 16S rRNA gene sequencing, and to follow their pregnancy outcomes. If the AMA group differed from the normal group in the microbial-associated 16S rRNA genes, it could reflect an endogenous microbial difference between the two groups and support the hypothesis that the AF is not sterile; otherwise, the microorganisms could be generated by exogenous contamination, which could not rule out the possibility that AF is sterile.

**Fig. 1:**
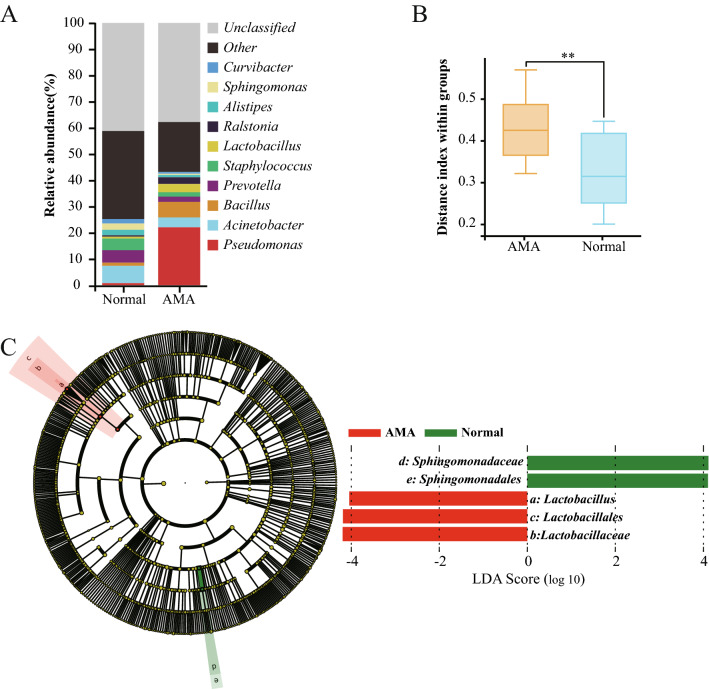
Differences between the normal and AMA AF samples in species composition and structure of communities. **A** Detected compositions and relative abundances of bacterial communities according to 16S rRNA gene sequencing. The normal age (left) and AMA (right) bacterial relative abundances of the top 10 bacterial communities were labeled with different colors. Unclassified: unclassified bacterial communities; others: other bacterial communities. **B** Comparison of distance indexes indicating species composition and structure of communities between the normal and AMA groups. (Weighted_unifrac, Welch’s *t* test, *P * =  *0.017*). **C** Circular cladogram indicating the polygenetic distribution of the bacterial lineages in the AF samples from the normal and AMA groups as determined by the linear discriminant analysis (LDA) effect size (LEfSe) (right) and comparison of the LDA effect size of the significantly different bacterial taxa (left). The cladogram illustrates the phylogenetic relationships among the significantly different bacterial taxa. The dots in the center represent the OTUs at the phylum level, while the dots in the outer circle represent the OTUs at the species level. Coloring principles: species with no significant difference were uniformly colored yellow; species for biomarkers were colored according to the different groups, with red nodes indicating bacteria with important roles in the AMA group, and green nodes indicating bacteria with important roles in the normal group. The names of the species represented by a, b, c, d, and e in the figure are shown in the LDA score illustration on the right. Indicators were defined as those with LDA  >  4. The histogram shows the LDA scores computed for significantly abundant taxa between the normal and AMA groups. The histogram represents the most significantly abundant taxa between the two groups. AMA: bacterial groups in the AMA group; normal: bacterial groups in the normal group

The main findings were: (1) B-ultrasonography and karyotype findings of the fetuses were normal and no bacteria were found by cultivation; (2) all 148 microbial-associated proteins in the normal human AF proteome were found in the 16S rRNA gene sequencing database, in which *Bacillus*, *Mycobacterium*, and *Pseudomonas* accounted for  ~ 20%; (3) the bacterial richness of the AF samples showed no significant difference between the AMA and normal groups (Chao1 index, Welch’s *t *test, *P * =  0.540; Additional file 2); (4) a significant difference in the species composition and structure of communities in the AF samples was found between the normal and AMA groups (Fig. 1A, B, Weighted_unifrac, OUT, Welch’s *t* test, *P * =  0.017); (5) the newborns were healthy and had no allergic reactions up to 90 days (Table 1). Collectively, these data suggest that the normal AF is not sterile and that the species composition and structure of communities change in the AMA group, although the bacterial richness may be similar and have no effect on the babies’ health.

**Table 1:** Clinical information of the newborns

No.	Age	Apgar	Delivery modes	Gestational weeks	W/L (birth) (kg/cm)	W/L (90 days) (kg/cm)	Feeding	Allergy
YS01	29	10/10	Eutocia	38^+6^	3.0/50	7.0/61	PB	No
YS02	33	10/10	Cesarean	37^+5^	3.4/49	6.3/64	B	No
YS03	30	10/10	Eutocia	38^+3^	2.9/50	6.8/62	PB	No
YS04	28	10/10	Cesarean	38^+5^	3.4/50	6/61.5	B	No
YS06	41	10/10	Cesarean	39^+2^	3.4/50	10/63	B	No
YS07	40	10/10	Eutocia	39^+0^	3.5/50	6.5/61	PB	No
YS08	40	10/10	Eutocia	37^+5^	3.3/48	7.0/62	B	No
YS09	39	10/10	Eutocia	39^+5^	4.1/52	5.0/65	B	No
YS10	40	10/10	Eutocia	40^+3^	4.0/51	9.0/65	B	No

Advanced maternal age was defined as age  >  35 years

*W* weight; *L* length; *PB* partial breastfeeding; *B* breastfeeding

The differences between the AF samples from the normal and AMA groups with regard to bacterial species composition and structure of communities were as follows. Five bacterial operational taxonomic units (OTUs) were increased in the AMA AF samples: *Lactobacillus helveticus*, *Pediococcus acidilacticii*, *Pasteurella multocida*, *Bacillus* indicus, and *Bacteroides vulgatus*. We used the LEfSe (Linear discriminant analysis Effect Size) method to identify bacterial OTUs that were likely to explain most of the differences between the normal and AMA AF samples. The bacterial orders of OTUs differed between the two groups. *Sphingomonadales* were more abundant in the normal group, while *Lactobacillales* were more abundant in the AMA group (Fig. 1C, LDA scores  >  4, Additional file 3). The differences in OTUs mainly spanned two Orders, with the Families Lactobacillaceae (primarily *Lactobacillus helveticus*) and *Sphingomonadaceae* accounting for the majority of the differences. Collectively, there may be a low concentration of microorganisms in the normal human AF samples and significant differences between the normal and AMA AF samples.

In the present study, we found that microbial-associated proteins and 16S rRNA genes could be identified in human AF at a low concentration. Furthermore, the species composition and structure of communities differed significantly between the normal and AMA AF samples. Thus, we conclude that the microbial-associated 16S rRNA gene in human AF is real, rather than occurring through microbial contamination, and that the bacteria in AF differ between normal age and AMA pregnancies. We already know that AF neutrophils can phagocytize bacteria during intra-amniotic infection [14], but we do not know the function of bacteria in normal AF. Two possible hypotheses were proposed based on our results. First, we suggest that the AF is not sterile, but the level of microbiota may be very low and under the mother’s immune system control. Therefore, the microbial communities may be related to the establishment of fetal immune function. Immunoglobulins from the mother may help to control the number or activity of the bacteria, to precisely control and activate the fetal immune system, given that recent studies demonstrated the presence of microbes or microbial DNA in the placenta, amniotic fluid [15], and meconium. Furthermore, Florent et al. [9] found live microbes in human fetuses such as *Lactobacillus*, and suggested that the selective presence of live microbes in fetal organs may have broader implications toward the establishment of immune competency and priming before birth, with the microbial exposure priming fetal immune cells during early human development. Second, we suggest that the bacterial 16S rRNA gene may come from the mother, because Rodriguez and colleagues proved that bacteria are transferred to the fetus from the mother by testing the meconium of healthy babies [16]. In addition, circulating cell-free DNA fragments are able to transfer between the fetus and the mother [17], and the bacterial 16S rRNA gene can be successfully detected in cell-free plasma DNA [18]. In this way, the bacterial 16S rRNA gene could possibly enter the AF through the umbilical cord, and this would explain why we were able to identify the 16S rRNA gene and peptides, but could not cultivate the bacteria.

## Materials and methods

### AF sample collection and preparation

Human amniotic fluid samples (~ 10 ml) were obtained by amniocentesis from women at 18–22 weeks of gestation who were undergoing prenatal diagnosis due to AMA or noninvasive prenatal testing for high-risk pregnancy after receiving written informed consent. The samples were collected in an operating room with the help of ultrasonic guidance; the operating room was sterile and the surgical instruments underwent aseptic processing and packaging. Nine samples from chromosomally normal pregnancies were chosen randomly.

### 16S rRNA gene sequencing

Microbial DNA was extracted from AF samples using an E.Z.N.A. Stool DNA Kit (Omega Biotek, Norcross, GA, USA) according to the manufacturer’s protocol. The 16S rDNA V3–V4 region of the eukaryotic rRNA gene was amplified by PCR using the following thermal profile: 95 °C for 2 min, followed by 27 cycles of 98 °C for 10 s, 62 °C for 30 s, and 68 °C for 30 s, and a final extension at 68 °C for 10 min. The primers used were 341-F: 5′-CCTACGGGNGGCWGCAG-3′and 806-R: 5′-GGACTACHVGGGTATCTAAT-3′, where the barcode was an 8 bp sequence unique to each sample. The PCR amplifications were performed in triplicate using 50-μl mixtures containing 5 μl of 10 × KOD buffer, 5 μl of 2.5 mM dNTPs, 1.5 μl of each primer (5 μM), 1 μl of KOD polymerase, and 100 ng of template DNA.

Amplicons were extracted from 2% agarose gels and purified using an AxyPrep DNA Gel Extraction Kit (Axygen Biosciences, Union City, CA, USA) according to the manufacturer’s instructions, and quantified using a QuantiFluor-ST System (Promega, Madison, WI, USA). Purified amplicons were pooled in equimolar quantities and subjected to paired-end sequencing (2  ×  250) on an Illumina HiSeq 2500 Platform (Illumina Inc., San Diego, CA, USA) according to standard protocols.

### Reanalysis of human AF raw data

The wiff. MS data files from Liu et al. [11] were searched against the Swiss-Prot database (*Homo sapiens*, *Acidobacteria*, *Actinobacteria*, *Bacteroidetes*, *Chloroflexi*, *Cyanobacteria*, *Firmicutes*, *Gammaproteobacteria*, *Patescibacteria*, *Planctomycetes*, and *Verrucomicrobia* protein sequences, release 2020_06) [19] using MaxQuant software (version 1.3.0.5) [20]. False discovery rates (FDRs) were estimated using the target-decoy strategy, and the FDR cut-offs were set to 0.01 for sites, peptides, and proteins. Enzyme specificity was considered to be full cleavage by trypsin, and two maximum missed cleavage sites were permitted. The minimum required peptide length was set to 7 residues. Carbamidomethyl (C) and iTRAQ 8plex labels were set as fixed modifications. Variable modifications included oxidation (M) and acetylation (protein N-term).

### Statistical analysis

Bioinformatic analysis was performed using Omicsmart (http://www.omicsmart.com).

## Supplementary Information

**Additional file 1.** Human AF protein groups.

**Additional file 2.** Bacterial diversity indexes of the AF samples.

**Additional file 3.** LEfSe analysis of bacterial communities of the AF samples.

## Data Availability

The datasets used and/or analysed during the current study are available from the corresponding author on reasonable request.

## Abbreviation

**AF**: Amniotic fluid
